# Navigating Vaccines with Confidence: Assessing Current and Past Community-Based Vaccination Efforts in Rural Eastern North Carolina

**DOI:** 10.3390/vaccines14010021

**Published:** 2025-12-24

**Authors:** Sarah B. Maness, Alice R. Richman, Abby J. Schwartz, Leslie Sanchez

**Affiliations:** 1 Department of Health Education and Promotion, College of Health and Human Performance, East Carolina University, 300 Curry Court, Greenville, NC 27858, USA; 2 Narrative Arts, 226 N. Front Street, Wilmington, NC 28401, USA

**Keywords:** vaccine confidence, vaccine information, rural health, vaccine hesitancy, vaccine availability

## Abstract

**Introduction**: Vaccination has led to significant decreases in mortality over the last century but requires high levels of uptake to be successful in reducing a wide range of infectious diseases in communities. Vaccine hesitancy is on the rise in the United States with most adults not receiving all recommended vaccinations, and childhood vaccinations are declining. Living in a rural community with a lack of access to resources may further limit uptake of vaccines. Identifying strategies to enhance vaccine confidence and access may assist in increasing vaccine uptake. The purpose of this study was to describe the landscape of existing community-based vaccination efforts and assess the components of a successful community-based vaccination program in rural eastern North Carolina. To reach this purpose, we conducted qualitative interviews with those involved in running community-based vaccine education and navigation programming in rural eastern North Carolina.” **Methods**: Researchers conducted 25 semi-structured interviews with participants involved in vaccination efforts in three rural counties in Eastern North Carolina. Interviews were transcribed, coded in NVivo version 14, and analyzed using thematic analysis to synthesize insights from participants. **Results**: Study participants held manager or coordinator roles in area health agencies, clinics, and pharmacies. Reported strengths of current vaccine efforts in Eastern North Carolina included patient education, strong partnerships between organizations providing vaccines, and ability to engage community members. Successful vaccine efforts have engaged participants through social media, flyers, trusted leaders, and the provision of convenient vaccine appointments. Areas for improvement in vaccine efforts included engaging a wider audience at vaccine events, building trust in vaccines among community members, and expanding vaccine education for hard-to-reach populations. Participants supported the development of a proposed community-based education and navigation program and felt that community members would be interested. Identified challenges included low participation due to vaccine hesitancy which could be overcome through incentives and delivery from trusted individuals. **Conclusions**: We found that there is still a need for trust building, education, and patient engagement within the landscape of existing community-based vaccination efforts for vulnerable populations in rural eastern North Carolina. Study participants indicated support for the development of a vaccine education program and researchers determined the project to be feasible. Based on the results of this study, researchers developed and implemented an integrated vaccine education and navigation program in Eastern North Carolina.

## 1. Introduction

Lauded as public health’s greatest achievement, vaccination has led to dramatic decreases in morbidity and mortality from major diseases [1]. It is estimated that since 1974, vaccination has prevented 154 million deaths globally [2]. Confidence in vaccines is necessary for vaccine programs to achieve their goals, yet this confidence is declining [3]. In the United States (US), recent increases in the idea that vaccination is unsafe and unnecessary, known as vaccine hesitancy, has coupled with outbreaks of vaccine-preventable diseases such as measles and pertussis [4,5].

Vaccine hesitancy is context-specific and complex, can vary across time, places, and types of vaccines and is associated with lower vaccine uptake [6,7,8]. Most US adults have not received all recommended adult vaccines [9]. Additionally, childhood vaccines have declined in the United States since the COVID-19 pandemic [10]. At the beginning of the 2025 school year, vaccination coverage among kindergartners in the US decreased from the previous year for all reported vaccines (diphtheria, tetanus, and acellular pertussis (DTaP), measles, mumps and rubella (MMR), and polio [11].

In the US, vaccines are administered by healthcare professionals, often including pharmacists, in a range of settings. Medical settings are places such as doctors’ offices and health departments, while non-medical settings may include pharmacies, workplaces, schools, or mass vaccination sites [12]. Recent data indicates that more adult vaccines (COVID-19, influenza, HPV, pneumococcal, measles and Tdap) are given at pharmacies than in any other setting [13]. More than 90% of the US population lives within 5 miles of a pharmacy, making them a convenient setting for vaccination [14,15]. The cost of vaccination varies based on vaccine type and health insurance plan, which often cover vaccines recommended by the Advisory Committee on Immunization Practice [16]. Children without health insurance can receive routine vaccinations free of cost through the Center for Disease Control and Prevention’s Vaccines for Children Program.

Vaccine uptake has been found to be lower among rural, underserved populations in the US [17,18]. One study assessing both COVID-19 vaccine hesitancy and uptake by rurality found that rurality was a stronger indicator of low vaccine uptake than vaccine hesitancy [19]. This supports that living in a rural US community with a lack of access to resources may compound low uptake of vaccines [20].

The rural South is a key area in the US with demonstrated need for community-engaged approaches to identify barriers to and increase confidence in vaccination among vulnerable populations. Eastern North Carolina (ENC) is a rural, underserved region [21] of the US state of North Carolina. Of the counties with the highest percentages of families living in poverty in North Carolina, 8 of the top 10 most impoverished are in ENC [22]. COVID-19 vaccination uptake was lower in rural areas of North Carolina than in urban areas [23]. The current study focuses on Bertie, Hertford and Washington Counties. These three counties are poorer in comparison with the surrounding ENC 29 county subregion (ENC-29) [24]. These counties are indicated as Tier 1 counties, indicating most economically distressed and have a greater percentage of older and racial/ethnic minority residents below the federal poverty level in comparison with NC and ENC-29 [25].

Vaccine vaccination efforts are complex and must overcome not only community member vaccine hesitancy, but also system complexity. Efforts to increase vaccinations can include a wide array of strategies, such as community engagement, organizational partnerships, patient education, mobile clinics, or vaccine drives [26]. Vaccine navigation, defined as connecting people with vaccine resources difficult to obtain on their own, can also be vital to increasing vaccination rates [27]. Despite the documented rurality and economic distress in the ENC region, the current gaps in vaccine efforts are unclear.

Gaining a better understanding of what community-based vaccination efforts are currently available to help ENC residents access recommended vaccines is critical as such work supports a reduction in the risk of morbidity and mortality from vaccine-preventable diseases. There is little research specifically interviewing vaccine staff on their thoughts about improving vaccine efforts in rural areas. It is particularly important to understand the perspectives of staff working in vaccination in Eastern North Carolina to inform the development of additional programming to address unmet needs in the region. The purpose of this study was to describe the landscape of existing community-based vaccination efforts and assess the components of a successful community-based vaccination program in rural eastern North Carolina. To reach this purpose, we conducted qualitative interviews with those involved in running community-based vaccine education and navigation programming in rural eastern North Carolina.”

## 2. Materials and Methods

This study was approved by the Prevention Strategies Institutional Review Board. All participants consented to participate in the study.

### 2.1. Participants and Setting

Researchers conducted semi-structured qualitative interviews with individuals involved in community-based vaccination efforts in priority ENC counties, including Bertie, Herford, and Washington. These individuals could include, but were not limited to, clinic staff, administrators, clinicians, community health workers, social workers and other community leaders with involvement in community-based vaccination efforts. Inclusion criteria were having engaged in community-based vaccination efforts within the priority ENC counties, being 18 years or older, and speaking English. Exclusion criteria included individuals not involved in vaccination efforts within the three priority ENC counties, being younger than 18 years old, or those who do not speak English.

### 2.2. Measures

Researchers developed a topical guide for the semi-structured interviews, and a sample can be found in Appendix A. Interviews began by asking participants to describe their role in their organization and how they work with the community, followed by any current or past community vaccination efforts in their county. Participants were then asked a series of questions about these vaccine efforts, including what worked well, what strategies they used to engage people, and what could be improved. Next, participants were asked if money was not a constraint, how they would launch a community vaccination program and engage participants.

In the next part of the interviews, researchers shared plans to develop a community-based education and navigation program delivered by community health workers. A description of the program was provided to participants and they were then asked about their thoughts on the idea, whether their community members would be interested in a program, ideal locations to host the program, and potential challenges to implementing such a program. Before ending the interview, participants were asked if there were any other thoughts they would like to share.

Participants also completed a demographic survey that was administered by the interviewer. The demographic survey included four questions on gender, employment, race and Hispanic ethnicity. Participants were asked their employment status (Working full time, part time, multiple part-time jobs, not currently working, other, unknown or refused); description of race (White, Black or African American, American Indian or Alaska Native, Asian, Native Hawaiian and Pacific Islander, other); and Hispanic or Latino ethnicity (Yes, No). The demographic survey had an area for the interviewer to add two additional information points: the county where the interviewee is employed and the name of the employer.

### 2.3. Data Collection Procedures

Participants were recruited using purposive sampling based on internet searches for existing vaccine provision services in ENC, health department websites and emails to contacts at local Federally Qualified Health Centers that provided vaccines. If vaccine events were identified online, researchers contacted the associated organizations via email and phone and asked to interview event leaders. Researchers also called local pharmacies, senior centers, and existing contacts from previous projects in the area to see if they provided vaccinations and to understand programming and outreach if they did. The study team also used snowball sampling to ask those working in vaccine provision to identify others involved in community-based vaccination programming to participate. The study team included three female doctoral-level researchers and two female student researchers. All study team members live and work in ENC.

All interviews were conducted by phone or TEAMS/Webex. Three research members individually conducted interviews, including two senior researchers and two student researchers. Two senior team members trained the student researchers in qualitative interviewing and research methods. Interviewers read participants an overview of the study procedures, including data protections, study team contacts, and the voluntary nature of the study. Participant Researchers obtained verbal informed consent over the telephone from all participants prior to beginning the interviews. The study team member obtained consent and authorization, as well as signed and dated each informed consent form after marking whether participants verbally agreed or declined to participate in the study.

Interviews lasted 30–60 min and participants were given a $25 Visa gift card to thank them for their time upon completion of the interview. Participant confidentiality was maintained by reporting participant demographic data only in aggregate and storing audio recordings and transcripts in a password-protected cloud accessible only to study team members. The Prevention Strategies Institutional Review Board reviewed and approved all study procedures.

### 2.4. Qualitative Analyses

All interviews were audio-recorded and transcribed verbatim. Study team members first read through each transcript and developed a codebook using deductive codes from the interview guides and inductive codes based on review of the transcripts. The full codebook can be found in Appendix B. Two study team members then coded transcripts using NVivo qualitative analysis software version 14. One team member conducted coding for all manuscripts. To assess reliability, a second study team member analyzed a subset of 10 interviews.

The coders assessed inter-coder reliability using the Cohen’s kappa statistic calculated by NVivo software. Kappa values ranged from 0.62 to 0.98 indicating moderate to very strong levels of agreement. Coders met for consistency checks to discuss discrepancies and refine coding. Researchers then used thematic analysis to identify recurring patterns and ideas from the interviews. One researcher wrote thematic code summaries for key codes which were shared with all study team members for consensus. Researchers determined overarching themes by synthesizing insights based on patterns among the thematic code summaries.

## 3. Results

Twenty-five participants completed an interview. Nearly all participants were female (n = 23) and working full time (n = 23) (Table 1). No participants reported Hispanic/Latino ethnicity. In reporting race, 16 participants identified as White and 8 as Black or African American. Most participants were in a director, supervisor, management or coordinator role related to nursing, pharmacy, or area health agency.

Our results indicated that multiple vaccine efforts exist in ENC, and the greatest current strengths are providing educational materials at vaccine events and strong partnerships between organizations, such as event coordination between pharmacies and health departments. Engagement strategies are often through social media, flyers, trusted leaders and hosting convenient opportunities for vaccination. Areas to improve vaccine efforts included reaching a broader audience, increasing community trust in vaccines, and increasing attendance at vaccination events. If money were not a constraint, participants reported that they would focus efforts on engaging more patients via increased advertising and incentives.

Participants were in support of the development of a community-based education and navigation program and thought community members would be interested. However, potential challenges in implementing a community-based education program mentioned by participants include lack of participation due to vaccine hesitancy and lack of vaccine education. Participants shared that these potential challenges could be overcome through incentives and the involvement of trusted individuals. Detailed descriptions of these results and participant quotes are provided in the text and a summary of themes can be found in Table 2.

### 3.1. Existing Vaccine Efforts in Eastern North Carolina

The most common community vaccine efforts were providing educational materials to community members and patients followed by offering vaccine clinics. Participants also often mentioned outreach efforts to the community and creating partnerships with other organizations to increase vaccinations.

*“Let me tell you about vaccination efforts. As you know, we’ve done a lot of work with COVID prior to pandemic, during pandemic, and even post pandemic, as new therapeutics become available, making sure that the public understands how to access that. We’ve also had big initiatives with monkeypox. We’ve had initiatives with flu, RSV, all of the things around respiratory illnesses, vaccinations… Specifically, what we have done, we push out a lot of communications, meaning a lot of campaigns.”*

(Participant 6)

*“We do outreach events for churches, for you know communities like, you know, there’s like some gated communities, like retired kind of communities. That we go to those areas. We do like county, county staff will call, and we’ll set up something through the county like their, you know, all the county employees. You know, just anything and everything, if anybody asks, we do that.”*

(Participant 12)

### 3.2. Current Strengths in Vaccine Efforts

Participants found that patient education was a strength in current vaccine efforts. This could be through one-on-one conversations or outreach via social media or other advertising.

*“Honestly, just bringing it up, recommending things to those people. They just don’t realize, you know, that they may need a vaccine. So, just having that discussion with the patients really tends to help them and get people vaccinated.”*

(Participant 8)

*“I mean definitely the flow clinics and having like the outreach on social media, and doing the mailing, I think are a big thing. And even just people driving around and seeing the signs, you know, on the side of the road saying, come here, come get your vaccine from us, has really helped.”*

(Participant 3)

Additional strengths included having strong partnerships with other relevant organizations to collaborate on vaccine efforts.

*“I definitely think our strong partnerships and good relationships with our senior centers have been and will continue to be very beneficial. They’re working with their local entities, primarily health departments, to actual schedule onsite vaccine clinics. So, they’re a really good partner and resource. We have made connections with two pharmacies in [Redacted] County. One we’ve scheduled a clinic. The other we’re waiting. But just making sure that we’re accessing our long-term partnerships, which is really helpful.”*

(Participant 8)

### 3.3. Current Engagement Strategies for Vaccine Efforts

Common engagement strategies that participants discussed included social media, flyers, providing information from trusted leaders, and hosting convenient clinic hours and locations. Participants stated that ways to make clinics more convenient included having pop-up or mobile clinics within communities and offering after-hours vaccinations.

*“Yes, we did post on social media. We had fliers in the clinic. We handed out fliers. We, you know, encouraged patients to contact the clinic if they had questions. Although we did COVID vaccines at the regular appointment, we also had the flu clinics as well later in the evening, so patients were able to still to go to work and then come in after work to receive the vaccines.”*

(Participant 18)

*“So, we started with the computer center, of course, but understanding that not all adults go to senior centers we did a lot of outreaches through local churches. We did outreach through our Meals on Wheels program, our congregate nutrition program services as well. And then we also used social media a lot to put information out there. And because O’Neal’s is such a, you know, one of the main pharmacies ever in Washington County, we were able to schedule flu clinics or COVID shot clinics with them and promote in advance through fliers for like a month or two at a time. Distributing fliers at their pharmacy letting individuals know, older adults know when upcoming clinics were going to be.”*

(Participant 5)

### 3.4. Areas for Improvement in Vaccine Strategies

Areas that participants mentioned for improvement in vaccine efforts included reaching a wider audience, building trust in vaccines among vaccine-eligible community members, and expanding vaccine education for hard-to-reach populations.

*“I still feel like there was a segment of the community we weren’t able to effectively reach. Those are more in the outlying areas. So, I wish we had the funding to do a more a public campaign in terms of maybe billboards or print media or things like that and not just rely on social media and fliers. but unfortunately, we weren’t able to do that.”*

(Participant 5)

*“Also, I think I would have had more champions, particularly people from faith-based communities. I don’t feel like we engaged them as much as we should have. That’s one thing that I would have had a few more pastors because I think, you know, the word is golden if it comes from a faith-based leader.”*

(Participant 25)

### 3.5. Participant Ideas for Ideal Vaccine Efforts

Participants offered a wide variety of ideas for vaccine efforts if money were not a constraint. Most participants stated that they would offer more incentives and have more advertising about vaccinations through the media. Many participants said they would also provide more education, offer more events and patient transportation.

*“Some nice fancy fliers, radio spots, newspaper advertisement, some things like that if that money is no option because sometimes it can be quite pricey. But just like more coordinated media response and things like that. Transportation to bring people here because that is sometimes a barrier, is actually people getting to where they’re doing the vaccines at.”*

(Participant 20)

### 3.6. Support for a Community-Based Vaccine Education and Navigation Program

Participants overwhelmingly said that planning a community-based vaccine education and navigation program was a good idea. Interviews indicated that participants have positive thoughts about incentives and use of community health workers. Some participants did mention a concern about how to pitch the program to the community to get them involved.

*“Absolutely, one hundred percent. I think the greatest thing ever is the community health worker model. It works, it works, it works. I think it would be very successful. And I also had the opportunity to interview a couple of community health workers in some of our Latinx work and they’re known, they’re trusted. They can shift their vernacular and communication in a way that people in the community can understand and they can help rally to bring them to the table.”*

(Participant 6)

Most participants said that community members would be interested in a community-based vaccine education and navigation program, especially if there was an incentive.

*“For the working class, for the retired people, it’s rough for everybody and I know we work every day, we get a paycheck and it’s hard for us, so I think about the people that’s retired. They don’t get a whole lot of money in retirement um the people that you know that don’t work, I know for sure that $30 incentive would get them there. Also that you’re offering transportation to get these vaccinations is another big plus because working in the rural areas I knew that transportation is a barrier in these rural counties. So yeah, offering that monetary incentive and also offering transportation are going to be two big pluses to get people there.”*

(Participant 15)

Some participants mentioned low participation in past vaccine efforts, which may be ameliorated through incentives or the involvement of trusted individuals.

*“Well, initially my first thought is it’s very hard to get people out, especially in these three counties. But by your offering an incentive that may make it better. Because I know when the COVID19 vaccine was administrated they were given I think a twenty-five-dollar gift card for the people that were getting vaccinated and the people who were driving them. I mean the turnout was outrageous because they were after those gift cards. But since then, the number of people that want to get vaccinated has dropped. So, I think offering the incentive would help. I just don’t know how much it would help.”*

(Participant 7)

### 3.7. Potential Locations for a Community-Based Vaccine Education and Navigation Program

The most popular locations mentioned by participants for a community-based vaccine education and navigation program were churches, schools, health-related agencies, such as a health department or community health center. Some participants mentioned hosting events in the parking lots of a big box store or grocery store.

*“Well, like I said, a lot of people are in and out of Walmart where there’s a big parking lot we could set up a tent out there and talk about the importance of vaccination and trying to keep your children caught up on their vaccinations, and scheduling their appointments, you know, to their pediatrician or the health department to make sure their children stay on top of their vaccines.”*

(Participant 23)

Participants spoke about the rurality of their counties and expressed concern that they did not know many available places to host vaccine efforts.

*“You may run into the same thing because you have these people that all the counties are rural, but you’re got people in the deep country, and they are the hardest ones to reach. And then, like I said, staffing is always an issue because we just don’t have the staff to go house-to-house to see what people want to be vaccinated. But as far as [Redacted] County is concerned, I really can’t think of anywhere because all of the places that I know that have venues are like out on the outskirts.”*

(Participant 7)

### 3.8. Potential Challenges with a Community-Based Vaccine Education and Navigation Program

Participants reported potential challenges with a vaccine education program to be lack of participation, vaccine hesitancy, and low education about vaccines. Participants also shared their perception that the community has low levels of trust in vaccination.

*“It doesn’t feel like people trust you as much. So, it’s almost got to be their idea, even as much as we try to promote things or even required, there’s you know just a lot of mistrust that happened over the past two years. Not that you know I’m saying that anything should have been mistrusted, I’m just saying from you know oh, I heard this, I heard this. You know, it made a horn grow out the side of my head, now I’m going to have a horn growing out, you know. So, it’s just got to be the buy-in from the community.”*

(Participant 12)

### 3.9. Ideas to Overcome Potential Challenges

To address challenges in vaccine efforts, participants suggested providing incentives and having more presence in the community.

*“I think it’s just the buy in, but you offer incentives, I think that would be a great idea. Or, you know, even if you, you know, if y’all are talking about, depending on what season you’re talking about doing, you know, we have the basketball games, we have football games, softball games, you know, just putting the information out there. I think if you are out there, if there’s somebody present, you know, putting this information out there and you’re not just putting fliers up because, you know, I can walk by twenty fliers. But somebody actually handing me a flier I may take a look at it and, okay, this is coming for whatever, So, it’s just more or less being present and not just sitting the information down if that makes sense.”*

(Participant 9)

*“I would say again going back to meeting people in a different context. You know, not necessarily focusing on what the purpose is as vaccination, health educator, partnership, but giving them a chance because people have got to know who you are. If they see you and you’re genuine, and then you establish a rapport and they realize you’re trying to build trust, they’ll be a little bit more open to you.”*

(Participant 22)

## 4. Discussion

Overall, we found that the landscape of existing community-based vaccination efforts for vulnerable populations in rural ENC would benefit from increased support in vaccine education and navigation. Participants indicated that strengths in current vaccine efforts included strong community partnerships. A recent publication on lessons learned from 21 community-engaged COVID-19 vaccine acceptance programs in diverse, rural communities also found strong community partnerships to be key to success [28]. In the US, there are no strict policies on requiring all vaccines (e.g., HPV) for school entry or employment, which makes adherence to age-appropriate vaccination more difficult than in some other countries [29].

Despite existing efforts, our participants indicated that there is still a need for a broader reach in patient education on the safety and efficacy of vaccines to increase patient engagement. A recent meta-analysis on community engagement in vaccine promotion found that while it has the potential to increase vaccine confidence, engagement requires a “fit for purpose” approach rather than one size fits all [30]. This aligns with our efforts to understand the specific needs of ENC.

Our findings regarding the need for more resources, advertising, and participant incentives are consistent with the region’s documented economic needs [21]. It is notable that aspects of strengths in vaccine efforts (e.g., patient education) were also mentioned as areas of improvement, because of the perceived need to expand these efforts to a broader audience. Similarly, the perceived need for more transportation and clinics in locations more convenient for patients aligns with previous research findings that vaccine hesitancy can be compounded by lack of access in rural areas [19].

Vaccine hesitancy was mentioned as a reason for lack of community participation in previous vaccine efforts. Vaccine hesitancy is well documented in rural and underserved areas [18,31]. Previous research among clinical workers in rural Alaska and Idaho found perceived vaccine confidence to be undermined by lack of trust, complacency, and convenience [32]. These three factors align with our findings that vaccine efforts can be hindered by both personal hesitancy and barriers to access such as transportation.

Our study found that staff involved in vaccine provision in ENC indicate an openness to the creation of a new community-based vaccine education and navigation program and the perception that community members would also be interested. Participants felt that challenges in vaccine hesitancy and engagement could be overcome with strategies such as incentives. However, the literature on the use of incentives to increase vaccine coverage has had mixed success, while noting this may be context-specific [33,34]. An additional strategy that participants suggested to increase community interest in a vaccine education and navigation program included the involvement of trusted individuals. A systematic review of COVID-19 vaccination messengers among Black communities in the USA found the most trustworthy messengers to be personal connections, those with shared community or identity, primary care providers, social network contacts, and faith leaders [35].

This study is not without limitations. This study focused on staff perspectives and did not include vaccine recipients or other community members. As such, the staff member perspectives may not reflect the community. The aim was not to reflect the community ecology but to understand what vaccination efforts exist in the region and to galvanize the perspectives of those running these efforts on what successful programming looks like. Participants willing to engage in an interview about vaccine efforts may be more supportive of and positive towards vaccination than others. Participant and interviewer power dynamics may have influenced how participants responded to questions. Most participants were female, so potentially differing perspectives of male staff members are not explored. However, this demographic finding may reflect the local workforce, as nursing, social work, and social services roles are more likely to be filled by women [36,37]. This qualitative study was not designed to be generalizable and may not be representative of the larger population. Participants did not always specify what vaccines were provided in specific existing vaccine efforts. We are unable to determine which vaccine uptake efforts were specific to the COVID-19 pandemic and/or are no longer offered.

The staff who participated in our study work directly with marginalized populations in rural communities and have expertise in knowing what is effective in providing vaccine education and promoting vaccine uptake. Incorporating their views should be considered best practices in working with rural communities and included in the development of programs aimed at increasing vaccine uptake. While existing health behavior theories that have been used to explain vaccine hesitancy, such as the Health Belief Model, focus on individual-level behaviors, our work also seeks to understand community-level issues in vaccination [38]. Our findings reflect the need for more than just education for behavior change; we need resources, partnerships and engagement as well.

Based on the findings of this study, researchers developed a vaccine education and navigation program that has been launched across three counties in ENC. The Navigating Vaccines Confidently community-based vaccination program took insights from this preliminary work to tailor the program to include incentives to increase participation and program delivery by Community Health Workers (CHWs) who are trusted individuals within the ENC counties. The vaccination program educates community members on age-appropriate vaccines, provides local resource guides where people can obtain vaccines, and offers navigation services to vaccination sites if needed. Navigation services delivered by CHWs include help scheduling an appointment for a vaccine, help with transportation (e.g., gas gift card provision), help with interpretation when getting a vaccine, meeting the person at a vaccine appointment for support or to help to complete paperwork, and/or help with childcare to attend a vaccine appointment. Future research will include the evaluation findings of the newly implemented program.

## 5. Conclusions

Overall, we found that there is still a need for trust building, education, and patient engagement within the landscape of existing community-based vaccination efforts for vulnerable populations in rural eastern North Carolina. Study participants indicated support for the development of a vaccine education program and researchers determined the project to be feasible. Our study contributes to the literature on vaccine efforts in rural areas by including the perceptions of staff with direct knowledge of the process of implementing local vaccine efforts. Our study has implications for local health practices and can be directly translated to the development of vaccine efforts. This work highlights the importance of gaining local insights and context in rural areas prior to the expansion of vaccine efforts and may inform research in other areas of the US South.

## Figures and Tables

**Table 1:** Participant demographics.

Demographic Variable	Participants (N = 25) n (%)
Race	
White	16 (64)
Black or African American	7 (28)
Other	2 (8)
Ethnicity	
Hispanic or Latino	0 (0)
Gender	
Male	2 (8)
Female	23 (92)
Employment Status	
Working full-time	24 (96)
Working part-time	1 (4)

**Table 2:** Summary of Themes.

Theme	Summary of Theme
Existing vaccine efforts in ENC	Multiple vaccine efforts exist in ENC including vaccine clinics, the distribution of educational materials, outreach and partnerships across organizations.
Current strengths in vaccine efforts	The most mentioned strengths in vaccine efforts are patient education and strong community partnerships.
Current engagement strategies for vaccine efforts	Engagement strategies are often conducted through social media, flyers, trusted leaders and hosting convenient opportunities for vaccination
Areas for improvement in vaccine strategies	Common areas for improvement in vaccine strategies include patient education, trust building, and patient engagement
Participant ideas for ideal vaccine efforts	If money were not a constraint, participants stated they would focus efforts on engaging more patients via increased advertising and the inclusion of incentives.
Support for a community-based vaccine education and navigation program	Participants stated they are in support of the development of a community-based vaccine education and navigation program and largely think community members would be interested.
Potential locations for a community-based vaccine education and navigation program	Participants suggested locations such as churches, schools, and health-related centers as well as parking lots of large stores.
Potential challenges with a community-based vaccine education and navigation program	Challenges in implementing a community-based vaccine education and navigation program could include lack of participation due to vaccine hesitancy and lack of vaccine education.
Ideas to overcome potential challenges	Potential challenges could be overcome through incentives and the involvement of trusted individuals.

## Data Availability

The participants of this study did not give written consent for their data to be shared publicly. Due to the sensitive nature of the study and small sample size, data is not available to be shared.

## Appendix A. Sample Interview Guide

Topical Guide: Navigating with Vaccine Confidence

1. Tell me about your role in (organization) and how you work with the community?
2. Share with me any current or past community vaccination efforts in your county? For example, ways that you’ve disseminated vaccines and/or vaccine information to residents in your county.
  - IF NO efforts past or present, Are there reasons why you have not engaged in these types of efforts?
3. Given what you have shared, what worked well in those efforts you have described?
4. What were strategies you used to get people to engage in your vaccination efforts?
  (Probe: How did you get people there to the vaccination events? How did you get people to attend the vaccination events?)
5. What areas of these vaccination efforts do you think could be improved in the future?
6. If money was not a constraint, and you were to launch a community vaccination program or vaccination information campaign in your county, what do you think would work best to engage the community? How would you do this?
7. We are planning to develop a community-based education and navigation program delivered by community health workers. This would include content such as vaccine education and navigation to age-appropriate vaccination for those who are interested. For example, this could include assistance with scheduling appointments for vaccinations, transportation to appointments, and/or translation or interpretation services. Residents who attend educational sessions will be provided $30 for their participation.
  - What do you think of this idea? Please explain.
  - Would community members/residents be interested in such a program? Please explain.
8. Where would be the best locations in your county to host the educational and navigation sessions?
9. Are there challenges in implementing a program like this in your community that we should be aware of? If so, what would be the best ways for us to address such challenges?
10. Is there anything I have not asked you that you would like to add?

## Appendix B. Codebook

**Code Name**	**Description**	**Question**
01 Work Role	Participant’s work role and its link to community	Tell me about your role in (organization) and how you work with the community?
02 Vaccine Efforts	Participant describes community vaccine efforts in their county, past or present.	Share with me any current or past community vaccination efforts in your county? For example, ways that you’ve disseminated vaccines and/or vaccine information to residents in your county.
03 No efforts	Participant describes why they haven’t engaged in community vaccine efforts	IF NO efforts past or present, Are there reasons why you have not engaged in these types of efforts?
04 Worked well	Participant describes parts of community vaccine efforts that worked well.	Given what you have shared, what worked well in those efforts you have described?
05 Engagement Strategies	Participant describes strategies used to get participants to engage in community vaccine efforts.	What were strategies you used to get people to engage in your vaccination efforts? (Probe: How did you get people there to the vaccination events? How did you get people to attend the vaccination events?)
06 Improve Effort	Participant describes vaccination effort areas that could be improved.	What areas of these vaccination efforts do you think could be improved in the future?
07 Ideal Effort	Participant describes how they would run vaccine efforts if money was not a constraint.	If money was not a constraint, and you were to launch a community vaccination program or vaccination information campaign in your county, what do you think would work best to engage the community? How would you do this?
08 Thoughtson Idea	Participants describe thoughts of the idea to develop a community-based education and navigation program delivered by community health workers.	We are planning to develop a community-based education and navigation program delivered by community health workers. This would include content such as vaccine education and navigation to age-appropriate vaccination for those who are interested. For example, this could include assistance with scheduling appointments for vaccinations, transportation to appointments, and/or translation or interpretation services. Residents who attend educational sessions will be provided $30 for their participation. 1. What do you think of this idea? Please explain.
09 Community Interest	Participants describe whether community would be interested in a community-based education and navigation program delivered by community health workers.	Would community members/residents be interested in such a program? Please explain.
10 Location for program	Participant describes locations to host new program.	Where would be the best locations in your county to host the educational and navigation sessions?
11 Potential Challenges	Participants describe challenges towards implementing proposed program	Are there challenges in implementing a program like this in your community that we should be aware of?
12 Address Challenges	Participants describe how to address potential challenges towards implementing proposed program.	If so, what would be the best ways for us to address such challenges?
13 Additional Thoughts	Participants add any additional thoughts.	Is there anything I have not asked you that you would like to add?

## References

[1] Montero D.A., Vidal R.M., Velasco J., Carreño L.J., Torres J.P., Benachi O M.A., Tovar-Rosero Y.Y., Oñate A.A., O’Ryan M. (2024). Two centuries of vaccination: Historical and conceptual approach and future perspectives. Front. Public Health.

[2] Shattock A.J., Johnson H.C., Sim S.Y., Carter A., Lambach P., Hutubessy R.C., Thompson K.M., Badizadegan K., Lambert B., Ferrari M.J. (2024). Contribution of vaccination to improved survival and health: Modelling 50 years of the Expanded Programme on Immunization. Lancet.

[3] De Figueiredo A., Simas C., Karafillakis E., Paterson P., Larson H.J. (2020). Mapping global trends in vaccine confidence and investigating barriers to vaccine uptake: A large-scale retrospective temporal modelling study. Lancet.

[4] Hotez P.J., Nuzhath T., Colwell B. (2020). Combating vaccine hesitancy and other 21st century social determinants in the global fight against measles. Curr. Opin. Virol..

[5] Nguyen K.H., Srivastav A., Lindley M.C., Fisher A., Kim D., Greby S.M., Lee J., Singleton J.A. (2022). Parental vaccine hesitancy and association with childhood diphtheria, tetanus toxoid, and acellular pertussis; measles, mumps, and rubella; rotavirus; and combined 7-series vaccination. Am. J. Prev. Med..

[6] MacDonald N.E. (2015). Vaccine hesitancy: Definition, scope and determinants. Vaccine.

[7] Quinn S.C., Jamison A.M., An J., Hancock G.R., Freimuth V.S. (2019). Measuring vaccine hesitancy, confidence, trust and flu vaccine uptake: Results of a national survey of White and African American adults. Vaccine.

[8] Rane M.S., Kochhar S., Poehlein E., You W., Robertson M.M., Zimba R., Westmoreland D.A., Romo M.L., Kulkarni S.G., Chang M. (2022). Determinants and trends of COVID-19 vaccine hesitancy and vaccine uptake in a national cohort of US adults: A longitudinal study. Am. J. Epidemiol..

[9] Centers for Disease Control and Prevention (2022). Vaccination Coverage Among Adults in the United States, National Health Interview Survey. https://www.cdc.gov/adultvaxview/publications-resources/adult-vaccination-coverage-2022.html.

[10] Hill H.A. (2024). Decline in vaccination coverage by age 24 months and vaccination inequities among children born in 2020 and 2021—National Immunization Survey-Child, United States, 2021–2023. MMWR Morb. Mortal. Wkly. Rep..

[11] Centers for Disease Control and Prevention (2025). Vaccination Coverage and Exemptions Among Kindergarteners. https://www.cdc.gov/schoolvaxview/data/index.html.

[12] Centers for Disease Control and Prevention National and State-Specific Estimates of Settings Where Adults Received Influenza, Updated COVID-19 and RSV Vaccinations, 2023–2024 Respiratory Virus Season, United States. https://www.cdc.gov/vaccines/imz-managers/coverage/national-state-vaccination-estimates.html#:~:text=Nonmedical%20settings,-57.3%20(56.6%2D58.0&text=Abbreviations:%20CI=confidence%20interval.,age%20group%20(Table%202).

[13] IQVIA (2023). Trends in Vaccine Administration in the United States. https://www.iqvia.com/insights/the-iqvia-institute/reports-and-publications/reports/trends-in-vaccine-administration-in-the-united-states.

[14] Strand M.A., Bratberg J., Eukel H., Hardy M., Williams C. (2020). Community pharmacists’ contributions to disease management during the COVID-19 pandemic. Prev. Chronic Dis..

[15] Qato D.M., Zenk S., Wilder J., Harrington R., Gaskin D., Alexander G.C. (2017). The availability of pharmacies in the United States: 2007–2015. PLoS ONE.

[16] Centers for Disease Control and Prevention (2024). How to Pay for Vaccines. https://www.cdc.gov/vaccines-adults/recommended-vaccines/how-to-pay-adult-vaccines.html#:~:text=Private%20health%20plans%20are%20required%20to%20cover,Committee%20on%20Immunization%20Practice%20at%20no%20cost.

[17] Tsai Y., Lindley M.C., Zhou F., Stokley S. (2021). Urban-Rural Disparities in Vaccination Service Use Among Low-Income Adolescents. J. Adolesc. Health.

[18] Smith B., Farakh F., Hanif A., Tunio J.H., Tunio S.N.J. (2025). Rural-Urban Disparities in COVID-19 Vaccine Uptake and Associated Mortality and Cardiovascular Disease Outcomes in the United States. Vaccines.

[19] Soorapanth S., Cheung R., Zhang X., Mokdad A.H., Mensah G.A. (2023). Rural–urban differences in vaccination and hesitancy rates and trust: US COVID-19 trends and impact survey on a social media platform, May 2021–April 2022. Am. J. Public Health.

[20] McKeirnan K.C., Undeberg M.R., Zelenko S., Meratnia G. (2024). A Qualitative Analysis of Rural Community Vaccination Barriers During the COVID-19 Pandemic. Vaccines.

[21] (2025). Rural Health Information Hub. North Carolina. https://www.ruralhealthinfo.org/states/north-carolina.

[22] HD Pulse. National Institute on Minority Health and Health Disparities. North Carolina Poverty–Map. https://hdpulse.nimhd.nih.gov/data-portal/social/map?age=001&age_options=ageall_1&demo=00009&demo_options=poverty_3&race=00&race_options=race_7&sex=0&sex_options=sexboth_1&socialtopic=080&socialtopic_options=social_6&statefips=37&statefips_options=area_states.

[23] Sandborn H., Delamater P., Brewer N.T., Gilkey M.B., Emch M. (2024). The geography of COVID-19 vaccine completion by age in North Carolina, US. PLoS ONE.

[24] East Carolina University Regional Transformation Data. https://regional-transformation-data.ecu.edu/drive-east-data/.

[25] US Census Bureau (2021). Tables. https://data.census.gov/table?g=040XX00US37_050XX00US37015,370.

[26] Centers for Disease Control and Prevention (2024). Vaccine Access for All People. https://www.cdc.gov/vaccines/basics/vaccine-equity.html#:~:text=Reducing%20Barriers%20to%20Vaccination:%20*%20Education:%20Campaigns,who%20have%20no%20or%20limited%20health%20insurance.

[27] Berlinger N., Mirabella J.M. (2021). Working Around the System: Vaccine Navigators and Vaccine Equity. https://www.thehastingscenter.org/working-around-the-system-vaccine-navigators-and-vaccine-equity/.

[28] Evans D., Norrbom C., Schmidt S., Powell R., McReynolds J., Sidibe T. (2023). Engaging community-based organizations to address barriers in public health programs: Lessons learned from COVID-19 vaccine acceptance programs in diverse rural communities. Health Secur..

[29] Marks T., Vanderslott S. (2021). Which Countries Have Mandatory Vaccine Policies? Our World in Data. https://ourworldindata.org/childhood-vaccination-policies.

[30] Xie Y.J., Liao X., Lin M., Yang L., Cheung K., Zhang Q., Li Y., Hao C., Wang H.H., Gao Y. (2024). Community engagement in vaccination promotion: Systematic review and meta-analysis. JMIR Public Health Surveill..

[31] Doherty I.A., Pilkington W., Brown L., Billings V., Hoffler U., Paulin L., Kimbro K.S., Baker B., Zhang T., Locklear T. (2021). COVID-19 vaccine hesitancy in underserved communities of North Carolina. PLoS ONE.

[32] Robinson R., Nguyen E., Wright M., Holmes J., Oliphant C., Cleveland K., Nies M.A. (2022). Factors contributing to vaccine hesitancy and reduced vaccine confidence in rural underserved populations. Humanit. Soc. Sci. Commun..

[33] Schwalbe N., Hanbali L., Nunes M.C., Lehtimaki S. (2022). Use of financial incentives to increase adult vaccination coverage: A narrative review of lessons learned from COVID-19 and other adult vaccination efforts. Vaccine X.

[34] Wong J., Gill C., Abdo A., Eisa A. (2025). The influence of financial incentives on vaccination hesitancy: A narrative review of recent research. Vaccines.

[35] Rabin Y., Kohler R.E. (2025). COVID-19 vaccination messengers, communication channels, and messages trusted among Black communities in the USA: A review. J. Racial Ethn. Health Disparities.

[36] American Association of Colleges of Nursing (2024). Nursing Workforce Fact Sheet. https://www.aacnnursing.org/news-data/fact-sheets/nursing-workforce-fact-sheet.

[37] Kim J.J., Joo M.M., Curran L. (2023). Social work licensure: Earnings premium and gender disparity. J. Soc. Soc. Work. Res..

[38] Champion V.L., Skinner C.S. (2008). The health belief model. Health Behav. Health Educ. Theory Res. Pract..

